# Supplementary material for: A multi-hospital, clinician-initiated bacterial genomics programme to investigate treatment failure in severe Staphylococcus aureus infections
Source: Nat Commun. 2025 May 26;16:4869. doi: 10.1038/s41467-025-60045-4 (PMC12106732; doi:10.1038/s41467-025-60045-4)
Supplement: Supplementary file 1 — Supplementary Information [file 41467_2025_60045_MOESM1_ESM.pdf]

Figure S1. Definitions of persistent and recurrent infection.

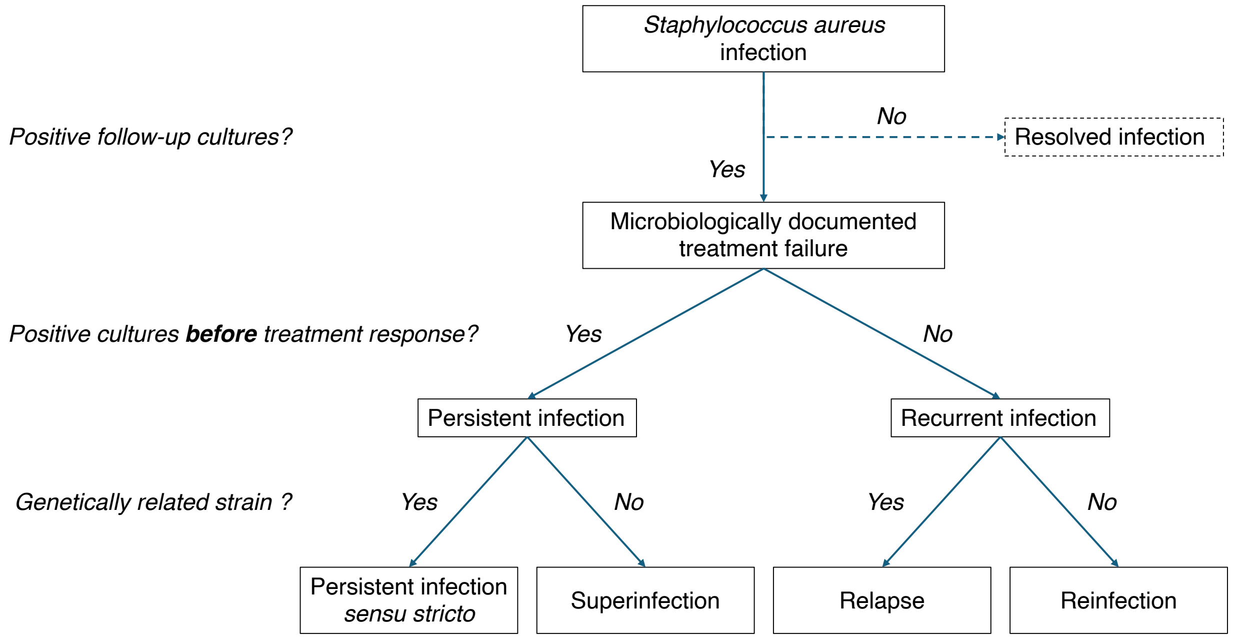

Figure S2. Genetic distance between 798 *S. aureus* isolates included in the global phylogeny (738 previously published Australian bacteraemia isolates and 60 isolates from 11 episodes of antibiotic failure included in this study). Pairwise distances (n=318,003) were calculated from the core genome alignment used to infer the phylogeny and from the core genome multi-locus sequence type analysis. The boxplots represent median, interquartile range (IQR) and 1.5 x IQR and are coloured according to 3 groups: isolates from the same host included in this study (within-host pairs), isolates from the external group (external pairs) and pairs involving one isolate from the current study and one from the external group (between-host pairs involving current study isolates). Panel A: normalised distance. Panel B: original distance based on raw data. Source data are provided as a Source Data file.

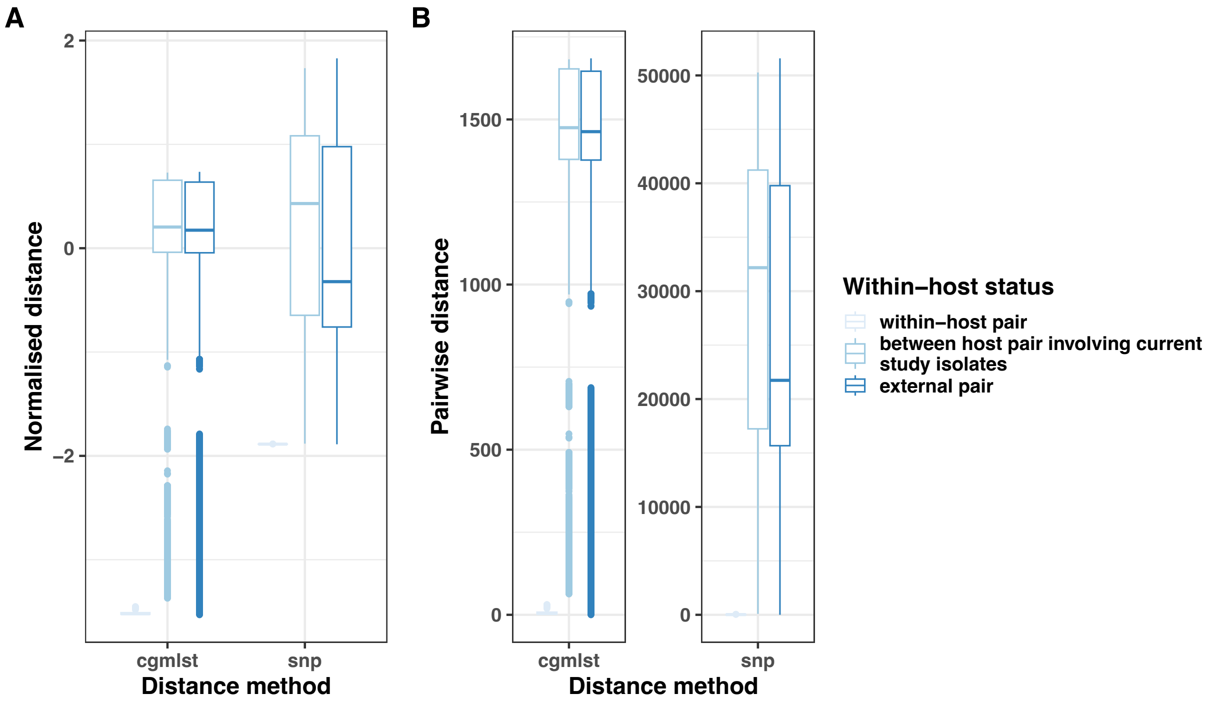

Table S1. Univariate and multivariate logistic regression of respondent recommendations for antibiotic switch and source control. OR: odds ratio; p: two-sided p values associated with the t statistic.

	Univariate		Multivariable	
**Predictor**	**OR**	**p**	**OR**	**p**
**Antibiotic switch**				
Adaptive mutations	3.76 (2.49-5.76)	5.14E-10	3.87 (2.55-5.97)	4.24E-10
Genomic report	0.61 (0.42-0.90)	1.15E-02	0.58 (0.39-0.86)	7.51E-03
Clinical microbiologist	0.76 (0.51-1.11)	1.54E-01	0.68 (0.40-1.16)	1.55E-01
S. aureus expertise	0.77 (0.53-1.12)	1.76E-01	0.72 (0.44-1.16)	1.81E-01
Genomic expertise	0.79 (0.53-1.18)	2.51E-01	1.14 (0.62-2.09)	6.74E-01
Country other than Australia	0.93 (0.62-1.39)	7.22E-01	0.95 (0.61-1.47)	8.12E-01
**Source control**				
Clinical microbiologist	0.21 (0.14-0.32)	1.95E-13	0.25 (0.14-0.44)	1.85E-06
S. aureus expertise	3.35 (2.27-4.98)	1.79E-09	7.28 (4.10-13.46)	5.44E-11
Genomic expertise	0.56 (0.38-0.84)	5.42E-03	0.42 (0.20-0.87)	2.00E-02
Country other than Australia	0.60 (0.40-0.90)	1.27E-02	0.28 (0.17-0.46)	5.47E-07
Genomic report	1.09 (0.75-1.59)	6.50E-01	1.13 (0.73-1.75)	5.99E-01
Adaptive mutations	1.08 (0.74-1.60)	6.85E-01	1.11 (0.71-1.75)	6.39E-01

Table S2. Univariate and multivariate linear regression of respondent recommendations for antibiotic duration. Beta: regression coefficient; p: two-sided p values associated with the t statistic.

	Univariate		Multivariable	
**Predictor**	**Beta**	**p**	**Beta**	**p**
Genomic expertise	0.34 (0.20-0.47)	1.59E-06	0.46 (0.27-0.65)	3.48E-06
Clinical microbiologist	0.18 (0.05-0.32)	7.07E-03	-0.08 (-0.24-0.09)	3.71E-01
Country other than Australia	-0.11 (-0.24-0.03)	1.35E-01	-0.07 (-0.20-0.07)	3.50E-01
Genomic report	0.07 (-0.06-0.20)	3.20E-01	0.06 (-0.06-0.19)	3.20E-01
Adaptive mutations	0.05 (-0.09-0.18)	4.88E-01	0.04 (-0.09-0.17)	5.06E-01
S. aureus expertise	-0.02 (-0.15-0.12)	8.20E-01	-0.18 (-0.33--0.03)	6.39E-01

Table S3. Description of clinical cases and genomic report provided to the RedCap survey participants.

Case label	Pre-genomics	Post-genomics
A/Jan 2019	*Recurrence of S. aureus (MSSA) bacteraemia 52 days after the initial episode.*	*Persistent infection*<br>*Adaptive mutations*<br><br>*Within-host evolution analysis of 1 baseline isolate and 3 recurrent isolates:*<br>*All isolates have an oxacillin MIC of 0.5 (broth microdilution) or 1 (E-test)*<br>*All isolates are S. aureus sequence type 15. Resistance genes identified are: blaZ.*<br>*We found 4 unique mutations among the 3 recurrent isolates. One mutation was found in rpoB. RpoB mutations have been associated with resistance to rifampicin, vancomycin, oxacillin and immune evasion.*
A/May 2019	*Persistent S. aureus (MSSA) bacteraemia over 7 days. Multiple infection foci: vertebral osteomyelitis, thigh abscess. Last positive sample after 9 days (paraspinal muscle). Antibiotic regime after 9 days: flucloxacillin, clindamycin, rifampicin.*	*Persistent infection*<br><br>*Within-host evolution analysis of 1 baseline isolate and 20 additional isolates:*<br>*All isolates are S. aureus sequence type 30. Resistance genes identified are: blaZ.*<br>*We found 3 unique mutations among the 20 additional isolates. No mutation was found in adaptive genes.*
C/Nov 2019	*Persistent S. aureus bacteraemia with right-sided endocarditis. Duration of bacteraemia 19 days. Recurrence 74 days after initial episode. High oxacillin MIC (Vitek) and small colony variants.*	*Persistent infection*<br>*Oxacillin MIC increase*<br>*Adaptive mutations*<br><br>*Within-host evolution analysis of 1 baseline isolate, 13 persistent isolates and 2 recurrent isolates:*<br>*Oxacillin MIC increased from 1.5 to 128 (E-test).*<br>*All isolates are S. aureus sequence type 188. Resistance genes identified are: blaZ.*<br>*We found 22 unique mutations among the additional isolates (recurrent and persistent). Mutated genes include: gdpP, clpX, pbp3 (associated with oxacillin resistance), stp1 (vancomycin resistance), rpoB (rifampicin, oxacillin, vancomycin*

		resistance, immune evasion); agrA (persistence).
G/Mar 2021	*S. aureus bacteraemia (MSSA) with vertebral osteomyelitis. Initial bacteraemia duration: 6 days. Recurrence of bacteraemia on cefazolin 31 days after the initial episode.*	*Persistent infection*<br><br>*Within-host evolution analysis of 1 baseline isolate and 1 recurrent isolate:*<br>*No cefazolin inoculum effect was found.*<br>*All isolates are S. aureus and belong to sequence type 5. Resistance genes identified are: blaZ.*<br>*We found 1 mutation. No mutation was found in adaptive genes.*
A/Aug 2021	*Recurrence of S. aureus (MSSA) bacteraemia 70 days after the initial episode.*	*Persistent infection*<br><br>*Within-host evolution analysis of 1 baseline isolate and 1 recurrent isolates:*<br>*All isolates are sequence type 88.*<br>*Resistance genes identified are: blaZ.*<br>*We found 3 mutations. No mutation was found in adaptive genes.*
C/Jul 2021	*Recurrence of S. aureus (MSSA) bacteraemia 91 days after the initial episode, one week after completing a course of cefazolin, followed by cephalexin (combined with ciprofloxacin for a week).*	*Persistent infection*<br>*Cefazolin inoculum effect*<br>*Adaptive mutations*<br><br>*Within-host evolution analysis of 1 baseline isolate and 1 recurrent isolate:*<br>*A cefazolin inoculum effect was found.*<br>*All isolates are S. aureus and belong to sequence type* 8. *Resistance genes identified are: blaZ.*<br>*We found 2 mutations, including in parC (associated with fluoroquinolone resistance).*
F/Oct 2021	*Recurrence of S. aureus (MSSA) bacteraemia 84 days after the initial episode.*	*Persistent infection*<br><br>*Within-host evolution analysis of 1 baseline isolate and 1 recurrent isolate:*<br>*All isolates are S. aureus and belong to a novel sequence type. Resistance genes identified are: blaZ, dfrG (trimethoprim), fusC (fusidic acid).*<br>*We found 1 mutation. No mutation was found in adaptive genes.*
A/Sep 2021	*S. aureus (MSSA) bacteraemia and vertebral osteomyelitis*	*Persistent infection*<br>*Oxacillin MIC increase*<br>*Adaptive mutations*

	with recurrence needing surgical revision. Isolation of strains with increased oxacillin MIC (4 mg/l).	*Within-host evolution analysis of 1 baseline isolate and 1 recurrent isolate:*<br>*Oxacillin MIC increased from 0.5 to 4 (broth microdilution).*<br>*All isolates are S. aureus sequence type 25.*<br>*Resistance genes identified are: blaZ.*<br>*We found 7 mutations. Mutated genes include: gdpP, fmtA (associated with oxacillin resistance), stp1 (vancomycin resistance).*
E/Apr 2022	*S. aureus (MSSA) bacteraemia and sternal infection complicated by 2 weeks bacteraemia.*	*Persistent infection*<br><br>*Within-host evolution analysis of 1 baseline isolate and 1 persistent isolate:*<br>*Both isolates have an oxacillin MIC of 1 (broth microdilution).*<br>*All isolates are S. aureus sequence type 88.*<br>*Resistance genes identified are: blaZ.*<br>*We found 2 mutations. No mutation was found in adaptive genes.*
D/Aug 2022	*Recurrence of S. aureus (MSSA) bacteraemia and vertebral osteomyelitis 106 days after the initial episode and breakthrough bacteraemia on antibiotic treatment 30 days after recurrence.*	*Persistent infection*<br><br>*Within-host evolution analysis of 1 baseline isolate, 1 recurrent isolate and 1 breakthrough isolate:*<br>*All isolates have an oxacillin MIC of 1 (broth microdilution), daptomycin MIC <= 0.25, vancomycin MIC <= 0.25.*<br>*All isolates are S. aureus and belong to a novel sequence type. Resistance genes identified are: blaZ.*<br>*We found 1 mutation. No mutation was found in adaptive genes.*
A/Mar 2023	*Breakthrough S. aureus bacteraemia and vertebral osteomyelitis 14 days after initial episode (on penicillin). Initial strain phenotypically penicillin susceptible, recurrent strain penicillin resistant.*	Persistent infection<br><br>*Within-host evolution analysis of 1 baseline isolate, 1 additional isolate and 2 breakthrough isolate:*<br>*All isolates have an oxacillin MIC of 1 (broth microdilution), daptomycin MIC <= 0.25, vancomycin MIC <= 0.25.*<br>*All isolates are S. aureus and belong to a novel sequence type. Resistance genes identified are: fosB (fosfomycin).*<br>*We found 0 mutations.*
