# Supplementary material for: A multi-hospital, clinician-initiated bacterial genomics programme to investigate treatment failure in severe Staphylococcus aureus infections
Source: Nat Commun. 2025 May 26;16:4869. doi: 10.1038/s41467-025-60045-4 (PMC12106732; doi:10.1038/s41467-025-60045-4)
Supplement: Supplementary file 2 — Reporting summary [file 41467_2025_60045_MOESM2_ESM.pdf]

nature portfolio

Corresponding author(s): Stefano G. Giulieri

Last updated by author(s): Apr 22, 2025

Reporting Summary

Nature Portfolio wishes to improve the reproducibility of the work that we publish. This form provides structure for consistency and transparency in reporting. For further information on Nature Portfolio policies, see our Editorial Policies and the Editorial Policy Checklist.

Statistics

For all statistical analyses, confirm that the following items are present in the figure legend, table legend, main text, or Methods section.

n/a	Confirmed	
☐	☒	The exact sample size (*n*) for each experimental group/condition, given as a discrete number and unit of measurement
☐	☒	A statement on whether measurements were taken from distinct samples or whether the same sample was measured repeatedly
☐	☒	The statistical test(s) used AND whether they are one- or two-sided<br>*Only common tests should be described solely by name; describe more complex techniques in the Methods section.*
☐	☒	A description of all covariates tested
☒	☐	A description of any assumptions or corrections, such as tests of normality and adjustment for multiple comparisons
☐	☒	A full description of the statistical parameters including central tendency (e.g. means) or other basic estimates (e.g. regression coefficient) AND variation (e.g. standard deviation) or associated estimates of uncertainty (e.g. confidence intervals)
☐	☒	For null hypothesis testing, the test statistic (e.g. *F*, *t*, *r*) with confidence intervals, effect sizes, degrees of freedom and *P* value noted<br>*Give P values as exact values whenever suitable.*
☒	☐	For Bayesian analysis, information on the choice of priors and Markov chain Monte Carlo settings
☒	☐	For hierarchical and complex designs, identification of the appropriate level for tests and full reporting of outcomes
☒	☐	Estimates of effect sizes (e.g. Cohen's *d*, Pearson's *r*), indicating how they were calculated

Our web collection on statistics for biologists contains articles on many of the points above.

Software and code

Policy information about availability of computer code

Data collection	RedCap 15.0.13
Data analysis	Kraken v2.1.1<br>Shovill v1.0.1<br>SPAdes v3.14.1<br>Prokka v1.14.6<br>Mlst v2.19.0<br>Abricate v1.0.1<br>Snippy v4.6.0<br>Snippy-core v4.6.0<br>Goalign v0.3.4<br>SNP-sites v2.5.1<br>IQ-TREE v2.0.3<br>ggtree v3.12.0<br>SNP-dists v0.7.0<br>chewBBACA v3.3.10<br>cgmlst-dists v0.4.0<br>R v4.4.1<br>All code and scripts are deposited on Zenodo (DOI: 10.5281/zenodo.15258746)

For manuscripts utilizing custom algorithms or software that are central to the research but not yet described in published literature, software must be made available to editors and reviewers. We strongly encourage code deposition in a community repository (e.g. GitHub). See the Nature Portfolio guidelines for submitting code & software for further information.

Data

Policy information about availability of data

All manuscripts must include a data availability statement. This statement should provide the following information, where applicable:

- Accession codes, unique identifiers, or web links for publicly available datasets
- A description of any restrictions on data availability
- For clinical datasets or third party data, please ensure that the statement adheres to our policy

Sequence data that support the findings of this study have been deposited in NCBI with the accession code PRJEB85744.

Research involving human participants, their data, or biological material

Policy information about studies with human participants or human data. See also policy information about sex, gender (identity/presentation), and sexual orientation and race, ethnicity and racism.

Reporting on sex and gender	Sex and gender was not considered in the study design and were not collected. This was a pilot genomic study and we decided to limit the clinical information to the minimum necessary to support the interpretation of antibiotic failure.
Reporting on race, ethnicity, or other socially relevant groupings	Not applicable
Population characteristics	As this was a pilot laboratory study, no demographic or comorbidities data were collected.
Recruitment	Participants recruitment was based on referral for bacterial whole-genome sequencing by the treating infectious diseases clinicians and/or the clinical microbiologists. As such, a selection bias might have emerged towards more complex cases and/or cases with in vitro phenotypic changes.
Ethics oversight	Austin Health Human Research Ethics Committee (HREC/105787/Austin-2024), Melbourne, Australia. Waiver of consent was provided by the Ethics Committee.

Note that full information on the approval of the study protocol must also be provided in the manuscript.

Field-specific reporting

Please select the one below that is the best fit for your research. If you are not sure, read the appropriate sections before making your selection.

☒ Life sciences ☐ Behavioural & social sciences ☐ Ecological, evolutionary & environmental sciences

For a reference copy of the document with all sections, see nature.com/documents/nr-reporting-summary-flat.pdf

Life sciences study design

All studies must disclose on these points even when the disclosure is negative.

Sample size	This is a pilot study with 11 participants. No sample size calculation was performed.
Data exclusions	No data was excluded.
Replication	The findings were not reproduced in another cohort. We are planning a larger study.
Randomization	Not relevant for this study as there was no group allocation.
Blinding	Not relevant for this study as there was no group allocation.

Reporting for specific materials, systems and methods

We require information from authors about some types of materials, experimental systems and methods used in many studies. Here, indicate whether each material, system or method listed is relevant to your study. If you are not sure if a list item applies to your research, read the appropriate section before selecting a response.

Materials & experimental systems

n/a	Involved in the study
☒	☐ Antibodies
☒	☐ Eukaryotic cell lines
☒	☐ Palaeontology and archaeology
☒	☐ Animals and other organisms
☐	☒ Clinical data
☒	☐ Dual use research of concern
☒	☐ Plants

Methods

n/a	Involved in the study
☒	☐ ChIP-seq
☒	☐ Flow cytometry
☒	☐ MRI-based neuroimaging

Clinical data

Policy information about clinical studies

All manuscripts should comply with the ICMJE guidelines for publication of clinical research and a completed CONSORT checklist must be included with all submissions.

Clinical trial registration	Not applicable.
Study protocol	Not applicable.
Data collection	This is a retrospective study including patients hospitalised between 2020 and 2023.
Outcomes	No predefined outcomes.

Plants

Seed stocks	*Report on the source of all seed stocks or other plant material used. If applicable, state the seed stock centre and catalogue number. If plant specimens were collected from the field, describe the collection location, date and sampling procedures.*
Novel plant genotypes	*Describe the methods by which all novel plant genotypes were produced. This includes those generated by transgenic approaches, gene editing, chemical/radiation-based mutagenesis and hybridization. For transgenic lines, describe the transformation method, the number of independent lines analyzed and the generation upon which experiments were performed. For gene-edited lines, describe the editor used, the endogenous sequence targeted for editing, the targeting guide RNA sequence (if applicable) and how the editor was applied.*
Authentication	*Describe any authentication procedures for each seed stock used or novel genotype generated. Describe any experiments used to assess the effect of a mutation and, where applicable, how potential secondary effects (e.g. second site T-DNA insertions, mosiacism, off-target gene editing) were examined.*
